# Supplementary material for: From sequence to enzyme mechanism using multi-label machine learning
Source: BMC Bioinformatics. 2014 May 19;15:150. doi: 10.1186/1471-2105-15-150 (PMC4229970; doi:10.1186/1471-2105-15-150)
Supplement: Additional file 2 — Java code of ml2db. Additional file ml2db_code.tar.gz contains the Java source code to run the multi-label machine learning experiments and save the results to database. The code’s Javadoc is included. [file 1471-2105-15-150-S2.zip › additional file 2/ml2db/ml2db/doc/main/ml2db/mulan/predict/PredictionsSaver.html]

PredictionsSaver


JavaScript is disabled on your browser.


- Overview
- Package
- Class
- Use
- Tree
- Deprecated
- Index
- Help

- Prev Class
- Next Class

- Frames
- No Frames

- All Classes

- Summary:
- Nested |
- Field |
- Constr |
- Method

- Detail:
- Field |
- Constr |
- Method


main.ml2db.mulan.predict

## Class PredictionsSaver

- java.lang.Object
- - main.ml2db.mulan.predict.PredictionsSaver

- ---

    

  ```
  public class PredictionsSaver
  extends java.lang.Object
  ```

  A class to create and save some machine learning predictions. TODO 1. for now
  only for cross-evaluation, to do: train and test 2. only works if the true
  labels are known, to do: if labels are unknown, do not emit the status (tp,
  tn etc.). Before running, make sure the predictions record test is myIsam:
  ALTER TABLE `test`.`prediction\_records` ENGINE = MyISAM ;

  Version:
  :   25 Mar 2013

  Author:
  :   ldf

- - ### Field Summary

    Fields

    | Modifier and Type | Field and Description |
    | --- | --- |
    | `static java.lang.String` | `FALSE_NEGATIVES` |
    | `static java.lang.String` | `FALSE_POSITIVES` |
    | `static java.lang.String` | `TRUE_POSITIVES` |
  - ### Constructor Summary

    Constructors

    Constructor and Description

    `PredictionsSaver(CrossExperimenter crossExper,
    mulan.data.MultiLabelInstances train,
    mulan.data.MultiLabelInstances test,
    mulan.classifier.MultiLabelLearner trainedLearner,
    boolean isCrossEvaluation,
    boolean saveTrueNegatives,
    int fold)`

    Predictor hooked into a cross-evaluation experiment.

    `PredictionsSaver(uk.ac.standrews.utils.main.database.DbManager resultsDbManager,
    int trainArffId,
    int testArffId,
    IdMultiLabelInstances fullTrainSet,
    IdMultiLabelInstances fullTestSet,
    mulan.data.MultiLabelInstances trainSet,
    mulan.data.MultiLabelInstances testSet,
    MulanLearner baseLearner,
    mulan.classifier.MultiLabelLearner trainedLearner,
    boolean saveTrueNegatives,
    int fold)`

    `PredictionsSaver(TrainTestExperimenter ttExpManager,
    mulan.data.MultiLabelInstances trainSet,
    mulan.data.MultiLabelInstances testSet,
    mulan.classifier.MultiLabelLearner trainedLearner,
    boolean saveTrueNegatives)`

    Predictor hooked into a train-test evaluation experiment.
  - ### Method Summary

    Methods

    | Modifier and Type | Method and Description |
    | --- | --- |
    | `java.lang.String` | `algorithm()` |
    | `int` | `experimentId()` |
    | `PredictionsExperimentTable` | `experimentTable()` |
    | `IdInstance` | `fromWekaToIdInstance(weka.core.Instance wekaInst)` Get an identified instance corresponding to this weka instance (same attributes) |
    | `int[]` | `generatePredictions(mulan.classifier.MultiLabelOutput multiLabelOutput, IdInstance instance, IdMultiLabelInstances baseData, boolean[] trueLabels)` |
    | `java.lang.String` | `getAlgorithmName()` |
    | `IdMultiLabelInstances` | `getBaseDataToBePredicted()` |
    | `uk.ac.standrews.utils.main.database.DbManager` | `getDbMan()` |
    | `PredictionsExperimentTable` | `getExperimentTable()` |
    | `java.lang.String` | `getFilePath()` |
    | `IdMultiLabelInstances` | `getFullTrainingSet()` |
    | `java.lang.String` | `getLogFilePath()` |
    | `PredictionsRecordTable` | `getPredictionsTable()` |
    | `java.util.Vector<uk.ac.standrews.utils.main.database.TableRow>` | `getPredRecordsFromDb()` Get prediction records from db |
    | `java.lang.String` | `getSavedPredictionsToString(java.util.TreeMap<java.lang.String,int[]> predictions)` For test: load saved predictions from database for inspection. |
    | `java.lang.String` | `getStartTime()` |
    | `int` | `getTestArffId()` |
    | `mulan.data.MultiLabelInstances` | `getTestSet()` |
    | `int` | `getTrainArffId()` |
    | `mulan.classifier.MultiLabelLearner` | `getTrainedLearner()` |
    | `mulan.data.MultiLabelInstances` | `getTrainSet()` |
    | `java.util.TreeMap<java.lang.String,int[]>` | `predict()` |
    | `PredictionsRecordTable` | `predictionsTable()` |
    | `int` | `saveExperimentToDb()` |
    | `boolean` | `saveTrueNegatives()` |
    | `void` | `setFold(int fold)` |
    | `java.lang.String` | `startTime()` |

    - ### Methods inherited from class java.lang.Object

      `equals, getClass, hashCode, notify, notifyAll, toString, wait, wait, wait`

- - ### Field Detail


    - #### FALSE\_NEGATIVES

      ```
      public static final java.lang.String FALSE_NEGATIVES
      ```

      See Also:
      :   Constant Field Values


    - #### FALSE\_POSITIVES

      ```
      public static final java.lang.String FALSE_POSITIVES
      ```

      See Also:
      :   Constant Field Values


    - #### TRUE\_POSITIVES

      ```
      public static final java.lang.String TRUE_POSITIVES
      ```

      See Also:
      :   Constant Field Values
  - ### Constructor Detail


    - #### PredictionsSaver

      ```
      public PredictionsSaver(CrossExperimenter crossExper,
                      mulan.data.MultiLabelInstances train,
                      mulan.data.MultiLabelInstances test,
                      mulan.classifier.MultiLabelLearner trainedLearner,
                      boolean isCrossEvaluation,
                      boolean saveTrueNegatives,
                      int fold)
      ```

      Predictor hooked into a cross-evaluation experiment. Since the
      predictions will be generated for folds of the training set, the mapping
      between weka instance and identified instance will be read from the
      original training set.

      Parameters:
      :   `crossExper` - a cross-evaluation experiment
      :   `train` -
      :   `test` -
      :   `trainedLearner` -
      :   `isCrossEvaluation` -
      :   `saveTrueNegatives` -
      :   `fold` -


    - #### PredictionsSaver

      ```
      public PredictionsSaver(uk.ac.standrews.utils.main.database.DbManager resultsDbManager,
                      int trainArffId,
                      int testArffId,
                      IdMultiLabelInstances fullTrainSet,
                      IdMultiLabelInstances fullTestSet,
                      mulan.data.MultiLabelInstances trainSet,
                      mulan.data.MultiLabelInstances testSet,
                      MulanLearner baseLearner,
                      mulan.classifier.MultiLabelLearner trainedLearner,
                      boolean saveTrueNegatives,
                      int fold)
      ```


    - #### PredictionsSaver

      ```
      public PredictionsSaver(TrainTestExperimenter ttExpManager,
                      mulan.data.MultiLabelInstances trainSet,
                      mulan.data.MultiLabelInstances testSet,
                      mulan.classifier.MultiLabelLearner trainedLearner,
                      boolean saveTrueNegatives)
      ```

      Predictor hooked into a train-test evaluation experiment. Since the
      predictions will be generated for the test set, the mapping between weka
      instance and identified instance will be taken from the test set, not
      from the training set.

      Parameters:
      :   `ttExpManager` - a train-test experiment manager
      :   `trainSet` -
      :   `testSet` -
      :   `trainedLearner` -
      :   `saveTrueNegatives` -
      :   `fold` -
  - ### Method Detail


    - #### algorithm

      ```
      public java.lang.String algorithm()
      ```


    - #### experimentId

      ```
      public int experimentId()
      ```


    - #### experimentTable

      ```
      public PredictionsExperimentTable experimentTable()
      ```


    - #### fromWekaToIdInstance

      ```
      public IdInstance fromWekaToIdInstance(weka.core.Instance wekaInst)
      ```

      Get an identified instance corresponding to this weka instance (same
      attributes)

      Parameters:
      :   `wekaInst` - a weka instance

      Returns:


    - #### generatePredictions

      ```
      public int[] generatePredictions(mulan.classifier.MultiLabelOutput multiLabelOutput,
                              IdInstance instance,
                              IdMultiLabelInstances baseData,
                              boolean[] trueLabels)
      ```


    - #### getAlgorithmName

      ```
      public java.lang.String getAlgorithmName()
      ```


    - #### getBaseDataToBePredicted

      ```
      public IdMultiLabelInstances getBaseDataToBePredicted()
      ```


    - #### getDbMan

      ```
      public uk.ac.standrews.utils.main.database.DbManager getDbMan()
      ```


    - #### getExperimentTable

      ```
      public PredictionsExperimentTable getExperimentTable()
      ```


    - #### getFilePath

      ```
      public java.lang.String getFilePath()
      ```


    - #### getFullTrainingSet

      ```
      public IdMultiLabelInstances getFullTrainingSet()
      ```


    - #### getLogFilePath

      ```
      public java.lang.String getLogFilePath()
      ```


    - #### getPredictionsTable

      ```
      public PredictionsRecordTable getPredictionsTable()
      ```


    - #### getPredRecordsFromDb

      ```
      public java.util.Vector<uk.ac.standrews.utils.main.database.TableRow> getPredRecordsFromDb()
      ```

      Get prediction records from db

      Parameters:
      :   `expId` - a prediction experiment id

      Returns:
      :   a string with all the prediction records existing in database for
          that experiment id


    - #### getSavedPredictionsToString

      ```
      public java.lang.String getSavedPredictionsToString(java.util.TreeMap<java.lang.String,int[]> predictions)
      ```

      For test: load saved predictions from database for inspection.

      Parameters:
      :   `predictions` -

      Returns:
      :   a comma separated string containing the predictions, the first
          column is the instance identifier.


    - #### getStartTime

      ```
      public java.lang.String getStartTime()
      ```


    - #### getTestArffId

      ```
      public int getTestArffId()
      ```


    - #### getTestSet

      ```
      public mulan.data.MultiLabelInstances getTestSet()
      ```


    - #### getTrainArffId

      ```
      public int getTrainArffId()
      ```


    - #### getTrainedLearner

      ```
      public mulan.classifier.MultiLabelLearner getTrainedLearner()
      ```


    - #### getTrainSet

      ```
      public mulan.data.MultiLabelInstances getTrainSet()
      ```


    - #### predict

      ```
      public java.util.TreeMap<java.lang.String,int[]> predict()
      ```

      Returns:
      :   a map with as key an instance identifier and as value an array
          with the table row identifiers of this instance's predictions.


    - #### predictionsTable

      ```
      public PredictionsRecordTable predictionsTable()
      ```


    - #### saveExperimentToDb

      ```
      public int saveExperimentToDb()
      ```


    - #### saveTrueNegatives

      ```
      public boolean saveTrueNegatives()
      ```


    - #### setFold

      ```
      public void setFold(int fold)
      ```


    - #### startTime

      ```
      public java.lang.String startTime()
      ```


- Overview
- Package
- Class
- Use
- Tree
- Deprecated
- Index
- Help

- Prev Class
- Next Class

- Frames
- No Frames

- All Classes

- Summary:
- Nested |
- Field |
- Constr |
- Method

- Detail:
- Field |
- Constr |
- Method
